# Differential Effects of Antibiotic Therapy on the Structure and Function of Human Gut Microbiota

**DOI:** 10.1371/journal.pone.0080201

**Published:** 2013-11-25

**Authors:** Ana Elena Pérez-Cobas, Alejandro Artacho, Henrik Knecht, María Loreto Ferrús, Anette Friedrichs, Stephan J. Ott, Andrés Moya, Amparo Latorre, María José Gosalbes

**Affiliations:** 1 Unidad Mixta de Investigación en Genómica y Salud del Centro Superior de Investigación en Salud Pública e Instituto Cavanilles de Biodiversidad y Biología Evolutiva de la Universitat de València, Valencia, Spain; 2 CIBER en Epidemiología y Salud Pública, Madrid, Spain; 3 Institute for Clinical Molecular Biology at the Christian-Albrechts University, Kiel, Germany; 4 Department for Internal Medicine, University Hospital Schleswig-Holstein, Campus Kiel, Kiel, Germany; Cairo University, Egypt

## Abstract

The human intestinal microbiota performs many essential functions for the host. Antimicrobial agents, such as antibiotics (AB), are also known to disturb microbial community equilibrium, thereby having an impact on human physiology. While an increasing number of studies investigate the effects of AB usage on changes in human gut microbiota biodiversity, its functional effects are still poorly understood. We performed a follow-up study to explore the effect of ABs with different modes of action on human gut microbiota composition and function. Four individuals were treated with different antibiotics and samples were taken before, during and after the AB course for all of them. Changes in the total and in the active (growing) microbiota as well as the functional changes were addressed by 16S rRNA gene and metagenomic 454-based pyrosequencing approaches. We have found that the class of antibiotic, particularly its antimicrobial effect and mode of action, played an important role in modulating the gut microbiota composition and function. Furthermore, analysis of the resistome suggested that oscillatory dynamics are not only due to antibiotic-target resistance, but also to fluctuations in the surviving bacterial community. Our results indicated that the effect of AB on the human gut microbiota relates to the interaction of several factors, principally the properties of the antimicrobial agent, and the structure, functions and resistance genes of the microbial community.

## Introduction

Throughout evolution mammals have established symbioses with microbial communities, which are located in different organs and tissues of the body such as skin, mucosa, or the gastrointestinal tract. The gut microbiota in humans is a particularly complex ecosystem with few dominant phyla (Firmicutes, Bacteroidetes, Proteobacteria and Actinobacteria) but show greater microbial diversity at lower taxonomic levels and a high functional redundancy [1], [2]. The gut microbiota seems to be host-specific and rather stable under non- or small perturbations [3] and is involved in a large number of host beneficial functions such as food processing, growth regulation of the intestinal epithelium, development of the immune system, or protection against pathogens [2], [4], [5]. Because of the essential role of the microbiota in host life, imbalances in the gut microbial community may have an important impact on human health. This is apparent in some intestinal pathologies such as inflammatory bowel diseases or antibiotic-associated diarrhea [6].

Systematic antibiotic (AB) therapy represents a major public health problem because gut microbiota may be transformed into a reservoir of antibiotic resistance genes, promoting the appearance of harmful resistant strains [7], [8], [9], [10]. It also suppresses some protective members of the resident microbiota promoting overgrowth of opportunistic pathogens such as *Clostridium difficile* [11]. Moreover, AB therapy disturbs the gut microbiota and, concomitantly, affects human physiology, for instance carbohydrate metabolism or immunity [7], [12].

Antibiotic features such as class, spectrum or pharmacological properties affect the gut microbiota in different ways [13]. In addition, host-associated factors such as diet, life history, genetic or health status, properties of the gut microbial ecosystem itself like resistance and resilience, or even the interplay between the microbiota and its host also have an effect on microbiota composition and function. All these factors can mask changes caused exclusively by antibiotics, representing a real challenge when it comes to understand microbiota responses. Most recent studies into the impact of antibiotics on the microbiota have focused on the emergence of resistant strains, but few have described their influence on the microbial community itself [14], [15], [16], [17], [18]. These latter surveys, using 16S rRNA gene sequencing, have shown that short and long-term AB courses affect diversity and biomass of the intestinal microbiota, with microbial composition resilience remaining deficient for long time after AB-treatment [15], [16], [17], [18]. By contrast, the functional impact of AB on the microbial ecosystem has been addressed less frequently [19].

The use of the meta-“omics” approaches (metagenomics, metatranscriptomics, metaproteomics) has provided deeper insights into microbial communities in different ecosystems [2], [20], [21], [22], [23]. A recent integrated analysis has provided a better understanding of the nature of the complex processes underlying the whole human gut microbiota and its responses during beta-lactamic-therapy [12].

In the present work we studied the effect of different antibiotics on the human gut microbiota by a follow-up study comparing microbial communities before, during and after AB therapy in four individuals. We analyzed the changes in composition of the total (16S rRNA gene) and active (16S rRNA transcripts) microbiota throughout treatment. Furthermore, the functional analysis of the total gene content of the community showed, for the first time, how the mode of action and the antimicrobial effect of AB affected the functional potential of the community. Finally, we described the dynamics of resistance genes (i.e. the resistome) throughout the study, paying particular attention to those that become resident after AB-therapy.

## Materials and Methods

### Ethics statement

The study was approved by the Ethics Committee of the Medical Faculty of the Christian-Albrechts University Kiel, Germany. Informed written consent was obtained from all patients involved in the study.

### Sample collection and AB treatment regimen

Fecal samples were collected from four patients (herein referred to as patient A, B, C and D) at the Department of Internal Medicine of the University Hospital Schleswig-Holstein, Campus Kiel, Germany (UK-SH). Patient A was treated with moxifloxacin (400 mg/day) for 13 days. Moxifloxacin is a fourth-generation synthetic fluoroquinolone antibacterial agent with a bactericidal effect inhibiting cell replication. AB treatment for patient B consisted of a combined therapy with penicillin G and clindamycin on the day of admission, and subsequently with clindamycin alone (3×300 mg/day) for seven days. This semi-synthetic derivative belongs to the lincosamide class exerting a bacteriostatic effect due to the inhibition of protein synthesis. For patient C, AB therapy was initiated with cefazolin (3×2 g/day) for seven days and continued with ampicillin/sulbactam (2×750 mg) for seven more days. Patient D received an amoxicillin (3×1000 mg/day) treatment. The antibiotics used for these two latter two patients belong to the beta-lactam class and have a bactericidal effect inhibiting cell envelope synthesis. Main features of patients and therapy are shown in Table 1.

**Table 1:** Main features of the follow-up study.

Patient	Antibiotic	Mode of action/ Antimicrobial effect	Pathology	Sampling date	Samples
A	Moxifloxacin	Cell replication inhibitor/ Bactericidal	Bronchitis, pneumonia	day0-before AB	A_before
				day3-during AB	A3_D
				day6-during AB	A6_D
				day10-during AB	A10_D
				day13-during AB	A13_D
				3 days after AB	A_after
B	Clindamycin	Protein synthesis inhibitor/ Bacteriostatic	Erysipelas	day0-before AB	B_before
				day2-during AB	B2_D
				day5-during AB	B5_D
				day6-during AB	B6_D
				28 days after AB	B_after
C	Cefazolin/ Ampicillin/ Sulbactam	Cell envelop synthesis inhibitor/ Bactericidal	Infection pacemaker	day0-before AB	C_before
				day3-during AB	C3_D
				day6-during AB	C6_D
				day10-during AB	C10_D
				3 days after AB	C_after
D	Amoxicillin	Cell envelop synthesis inhibitor/ Bactericidal	Chronic sinusitis maxillans	day0-before AB	D_before
				day3-during AB	D3_D
				7 days after AB	D_after

AB, antibiotic; D, during the treatment.

Fecal samples from patients (named A, B, C and D) were collected on the day of admission, before the antibiotic treatment (day 0), during and after AB therapy. In two cases (A and B), the last sample was taken 3 days after therapy, in the other two cases (B and D) the last sample was provided 7 and 28 days after treatment, respectively (Table1). Patients did not present any intestinal disorder. Samples were collected in sterile tubes and stored at −80°C until further processing.

### DNA extraction

Tubes containing fecal samples with sterile PBS (containing, per liter, 8 g of NaCl, 0.2 g of KCl, 1.44 g of Na2HPO4, and 0.24 g of KH2PO4 [pH 7.2]) were centrifuged at 1250 g and 4°C for 2 min to remove fecal waste. DNA was extracted from bacterial pellets using QIAamp® DNA Stool Kit (Quiagen) following the manufacturer's instructions. The product was concentrated by precipitation using 0.1 V of NaCl 3 M and 2 V of ethanol 100% and diluted in 75 µl of nuclease-free water. A standard agarose gel electrophoresis was run to check the integrity of DNA. The total DNA obtained was quantified with Nanodrop-1000 Spectrophotometer (Thermo Scientific) and with the QuantiT PicoGreen dsDNA Assay Kit (Invitrogen).

### Amplification of the 16S rRNA gene

For each sample a region of the 16S rRNA gene was amplified by polymerase chain reaction (PCR). The primers used were the universal E8F (5'-AGAGTTTGATCMTGGCTCAG-3') with adaptor A and 530R (5'-CCGCGGCKGCTGGCAC-3') with adaptor B using the sample-specific Multiplex Identifier (MID) for pyrosequencing. The amplified region comprises hyper-variable regions V1, V2 and V3. For each sample a 50 µl PCR mix was prepared containing 5 µl of Buffer Taq (10X) with 20 mM MgCl2, 2 µl of dNTPs (10 mM), 1 µl of each primer (10 mM), 0.4 µl of Taq Fast start polymerase (5 u/ µl), 39.6 µl of nuclease-free water and 1 µl of DNA template. PCR was run under the following conditions: 95° for 2 min followed by 25 cycles of 95° for 30 s, 52° for 1 min and 72° for 1 min and a final extension step at 72° for 10 min. The amplification process was checked by electrophoresis in agarose gel (1.4%). PCR products were purified using NucleoFast® 96 PCR Clean-Up Kit (Macherey-Nagel) and quantified with Nanodrop-1000 Spectrophotometer (Thermo Scientific) and with the QuantiT PicoGreen dsDNA Assay Kit (Invitrogen). The pooled PCR products were directly pyrosequenced.

### Total RNA extraction and double-strand cDNA synthesis

Total RNA was extracted from fecal samples using RiboPure™-Bacteria Kit (Ambion). DNase treatment was applied to remove traces of genomic DNA from the eluted RNA using the same kit. The integrity of RNA was checked by electrophoresis in agarose gel (0.8%). The efficiency of the DNase treatment was checked by amplifying each RNA sample by PCR. To retro-transcribe total RNA into single-stranded cDNA the High-Capacity cDNA Reverse Transcription Kit (Ambion) was used. To synthesize double-stranded cDNA, 7.5 µl of *Escherichia coli* ligase buffer (10X), 2 µl of dNTPs (10 mM), 0.2 µl of *E. coli* RNAse H (5 u/µl), 3 µl of *E. coli* DNA pol I (10 u/µl), 0.5 µl of *E. coli* ligase (10 u/µl) and 41.8 µl of nuclease-free water were added to each single-stranded cDNA sample. The mixture was placed in a Thermocycler at 15°C for 2 hours. Then, 2.5 µl of T4 DNA polymerase (3 u/µl) were added and kept at 15°C for 30 min. The metatranscriptome obtained thus was purified by precipitation and quantified using Nanodrop-1000 Spectrophotometer (Thermo Scientific) and the QuantiT PicoGreen dsDNA Assay Kit (Invitrogen). A standard agarose gel electrophoresis was run to check the integrity of double-stranded cDNA.

### Pyrosequencing

For each sample, the total DNA (metagenome), double-stranded cDNA and amplicons of the 16S rRNA gene were sequenced with a Roche GS FLX sequencer and Titanium chemistry in the company Life Sequencing (Valencia, Spain) and in the Center for Public Health Research (CSISP-FISABIO) (Valencia, Spain). We obtained an average of 58,928, 41,838 and 4,872 reads per sample, respectively.

### Taxonomic assignment of 16S rRNA amplicons

We have used the Ribosomal Database Project (RDP) pyrosequencing pipeline [24] to trim off the MID and primers and to obtain the taxonomic classification. Sequences with a phred quality score less than 20 (Q20) and short length (<250pb) were discarded. We considered only annotations that were obtained with a bootstrap value greater than 0.8, leaving the assignation at the last-well identified level and consecutive levels as unclassified (uc).

### Taxonomic assignment of 16S rRNA transcripts

Due to the procedure followed to obtain the metatranscriptome, the vast majority of transcripts belonged to ribosomal genes (16S and 23S). The 16S rRNA reads were obtained from the total cDNA by comparing the total reads against the Small Subunit rRNA Reference Database (SSUrdb) [25] with BLASTN [26] and an e-value of 10-^16^. All sequences with detected homology were considered as 16S rRNAs and used to evaluate the phylogenetic diversity of the active bacteria. The taxonomic classification was performed in the same way as the amplicons.

### Analysis of total and active microbiota

To study the phylogenetic structure of the bacterial community we applied two approaches that involved the 16S rRNA gene. The widely used analysis of 16S rRNA gene amplicons shows the composition of the total microbiota (16S rRNA gene). However, since the growing (active) bacteria contain more ribosomal RNA than latent or starved cells, studying the 16S ribosomal RNA transcripts enabled the active members of the microbiota to be identified (16S rRNA transcripts) [12], [22]. We calculated sample diversity of the throughout the treatment for total and active bacteria by applying three parameters: two richness estimators, Chao1 [27] and the abundance-based coverage (ACE) [28], and the Shannon index [29]. These estimators are implemented in package Vegan [30] under R software (http://cran.r-project.org) [31]. The biodiversity index and richness estimators were calculated after sub-sampling with the multiple_rarefactions.py script of QIIME to avoid the bias of the sequencing effort [32]. We used heat maps based on taxonomic composition to study the similarity between samples due to the relative abundance of each taxon using the Vegan library in the R software (http://cran.r-project.org) [30], [31]. Canonical correspondence analysis (CCA) was performed to determine the relation between the sample composition and the class and mode of AB-treatment. To statistically assess the effect of such factors on the bacterial composition a multivariate ANOVA based on dissimilarity tests (Adonis) was applied, as implemented in the package Vegan, R software (http://cran.r-project.org) [30], [31].

### Metagenomics: functional analysis

To eliminate reads that were artifact replicates of pyrosequencing, we used the 454 Replicate Filter Program [33] with the following parameters: sequence identity cutoff  =  1; length difference requirement  =  0; number of beginning base pairs to check  =  10. Unique reads were compared against the human genome using BLASTN [26] with an e-value of 10^−10^ in order to remove human sequences. To identify the sequences encoding the ribosomal gene 16S rRNA we compared the dataset against the Small Subunit rRNA Reference Database (SSUrdb) described in Urich et al. [25] using BLASTN [26] with an e-value of 10^−16^. Sequences that did not give homology were used to identify the reads corresponding to the ribosomal gene 23S rRNA by BLASTN [26] against the Large Subunit rRNA Reference Database (LSUrdb) described in Urich et al. [25] with an e-value of 10^−4^. Reads that matched with the LSUrdb were discarded. The remaining reads were compared to the NCBI-nr protein database using BLASTX [26] to identify the protein-coding genes. Taxonomic assignment was based on the output of BLASTX applying the lowest common ancestor (LCA) algorithm. Fasta files were used to identify the Open Reading Frames (ORFs) by applying the facility of Fraggenscan from the web server of metagenomic analysis (WebMGA) [34]. To annotate the functions of the predicted ORFs, we applied HMMER 3.0 program [35] against TIGRFAM database [36] using default parameters. To identify the genes involved in resistance to antibiotics, we compared the identified ORFs against the Antibiotic Resistance Genes Database by BLASTp [37] with an e-value of 10^−10^. We used the ShotgunFunctionalizeR package [38] in the R software http://www.R-project.org/ [31] for functional comparison of metagenomes. Specifically, we applied the testGeneCategories.dircomp function to compare the distribution of functional categories between groups of samples. The test is based on a Poisson model and compares each gene family of a higher functional category to decide if the category is statistically significant among two groups of samples [38].

### Data accession number

All sequences have been entered in the European Bioinformatics Institute database, under accession number ERP002192.

## Results

### Dynamics of total and active microbiota composition throughout therapy

**Figure 1:**
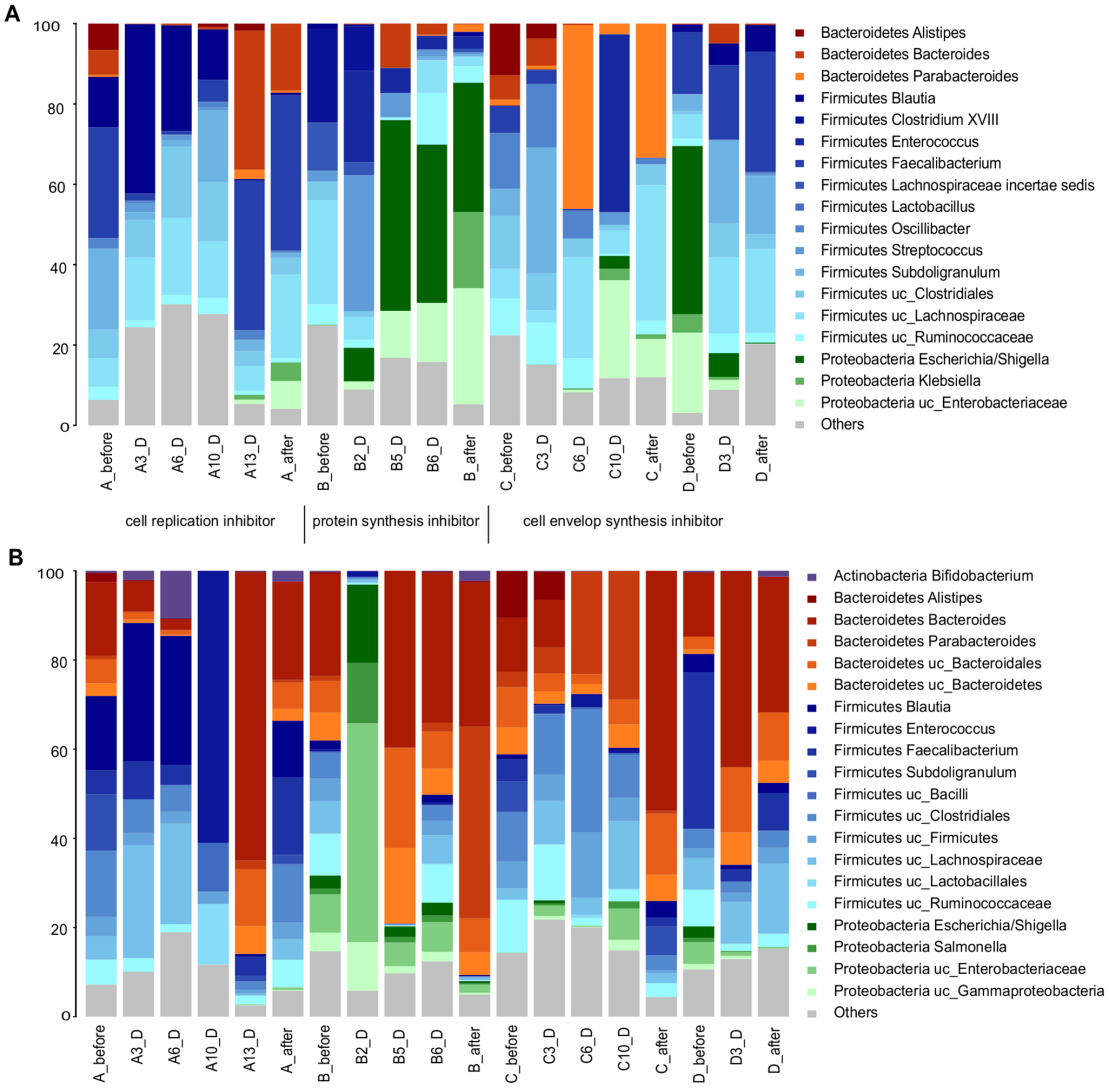
Microbiota composition of patients A, B, C, and D. (A) total microbiota (16S rRNA gene) (B) active microbiota (16S rRNA transcripts). The mode of action for each AB used is indicated.

We analyzed total (16S rRNA gene) and active (16S rRNA transcripts) microbiota from the four patients (A, B, C and D) throughout AB treatment. The antibiotics administered to patients A, C and D had a bactericidal antimicrobial effect, whereas in patient B the effect was bacteriostatic. Regarding the mode of action, the antibiotic used in patient A was a cell replication inhibitor, in patient B it was an inhibitor of protein synthesis, whereas patients C and D were treated with a cell envelop synthesis inhibitor (Table 1). Each patient not only presented their own microbiota profile for both total (Figure 1A) and active (Figure 1B) microbiota before treatment, but also there was apparently a rather large variation in bacterial taxa abundance during and after treatment, which we describe succinctly. In patient A, both total and active microbiota showed a high presence of the families Lachnospiraceae (*Coprococcus* and *Roseburia* genera) and Ruminococcaceae (*Faecalibacterium*, *Blautia* and *Subdoligranulum* genera) during AB treatment with fluoroquinolone (Figure 1). However, some genera such as *Faecalibacterium* and *Subdoligranulum* were negatively affected by the AB, while others such as *Blautia*, *Coprococcus*, *Coprobacillus* and *Collinsella* appeared to be resistant in the first stage of treatment. We also found that the bactericidal effect of AB had a negative impact on *Bacteroides* genus (Bacteroidetes phylum) in the first days of treatment, but the trend changed on day 13 with a great increase in its abundance. Treatment with clindamycin of patient B resulted in a high presence of Enterobacteriaceae (*Escherichia*, *Salmonella* genera), as shown in Figure 1B. We also observed an increase in the *Bacteroides* genus after the 5th day of treatment in active microbiota. For patient C, Oscillibacteriaceae and Ruminococcaceae families (Firmicutes phylum) as well as Rikenellaceae and Bacteroidaceae (Bacteroidetes phylum) constituted the most abundant taxa in the total microbiota (Figure 1A). The first important change occurred on day 6 with an increase in *Parabacteroides* (Bacteroidetes phylum), which remained abundant after treatment. However, in the active microbiota we observed a shift towards the Bacteroidetes phylum (*Alistipes* and *Bacteroides* genera) at the two last time points. On the 10th day of treatment, an increase in facultative anaerobic families, Enterobacteriaceae (Proteobacteria) and Enterococcaceae (Firmicutes) was found. Finally, in patient D, the initial microbiota composition consisted mainly of Enteriobacteriaceae (*Escherichia* genus) and Ruminococcaceae (*Faecalibacterium* genus). However, both genera were greatly affected by the antibiotic as there was an increase in resistant bacterial taxa of the *Bacteroides* genus (Bacteroidetes).

**Figure 2:**
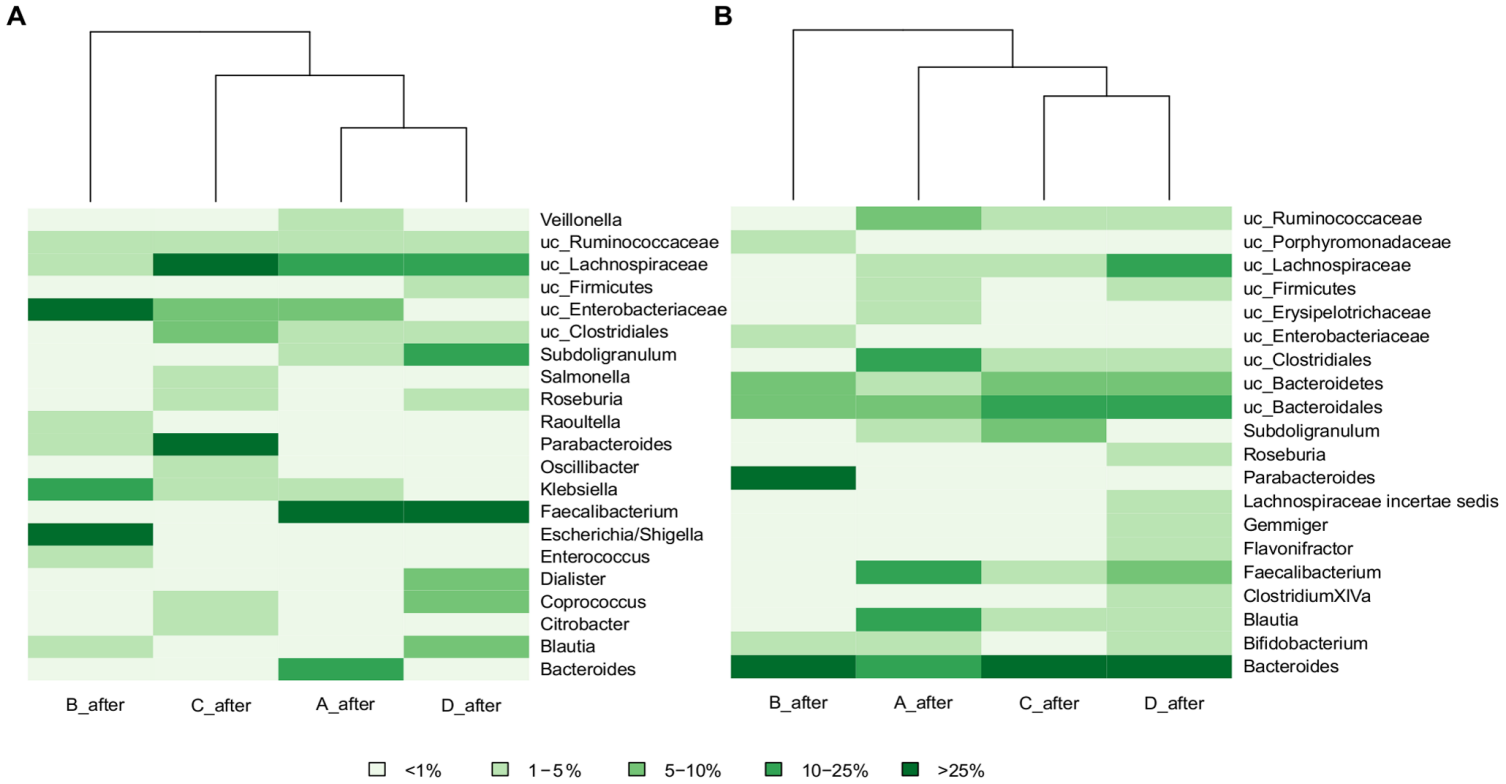
Heat map and clustering based on taxon composition and abundance. (A) total microbiota, (B) active microbiota. Colors in the figure depict the percentage range of sequences assigned to main taxa (abundance >1% in at least one sample).

After the AB course, patients A, C and D who received a bactericidal antimicrobial agent clustered together in both cases, total (Figure 2A) and active (Figure 2B) microbiota, apart from the patient treated with a bacteriostatic antibiotic (patient B) (Figure 1 and 2). Moreover, we observed that the two patients treated with cell envelope synthesis inhibitors (C and D) grouped together in the case of the active microbiota (Figure 2B).

For all patients, the diversity parameters (Shannon index, Chao1 and ACE estimators) of both total and growing microbiota, showed notable fluctuations with a decrease in the number of bacterial taxa and evenness on the first days of treatment (Figure S1). At the end of the AB course, these three biodiversity parameters increased but they did not reach the initial values observed before AB therapy (Figure S1).

### Effect of the class of antibiotic

**Figure 3:**
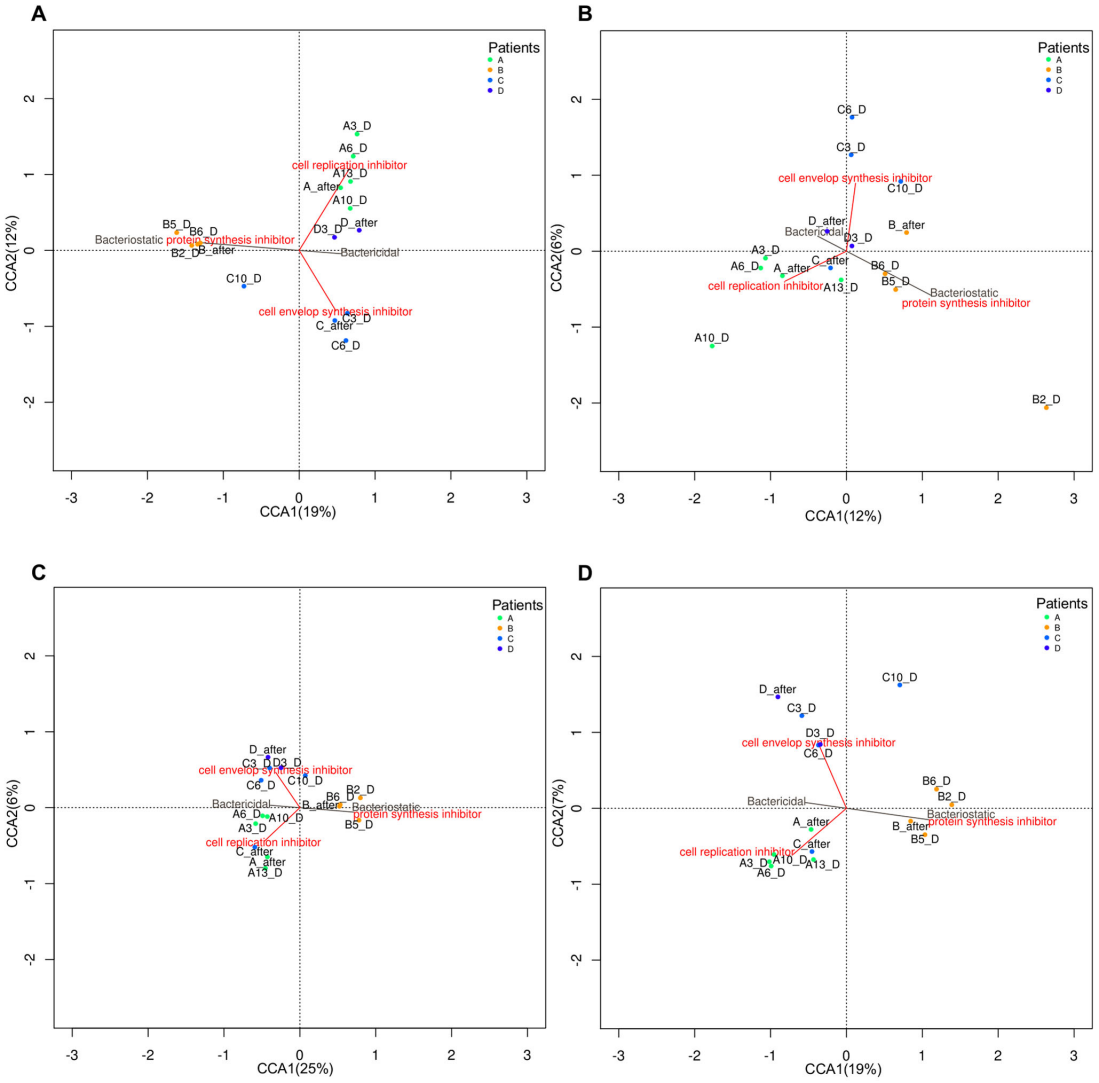
Canonical Correspondence Analysis (CCA) of the patients A, B, C and D in the follow-up study. (A) total microbiota, (B) active microbiota, (C) genes and (D) gene taxonomy. The antimicrobial effect is represented as a vector with two levels (bactericidal and bacteriostatic). The mode of AB action is represented as a vector with three levels (cell envelop synthesis inhibitor, cell replication inhibitor and protein synthesis inhibitor).

To evaluate the pattern of variation shown by bacterial taxa or gene abundances and its relationship with two variables (the antimicrobial effect -bactericidal and bacteriostatic- and the mode of action of the antibiotic -protein synthesis inhibitor, cell replication inhibitor and cell envelope synthesis inhibitor-) we applied a CCA at the different levels: 16S rRNA gene, 16S rRNA transcripts, genes and the taxonomy of the identified coding regions (gene taxonomy). The results showed that these two factors (antimicrobial effect and mode of action) accounted for variability in a particular direction and with different strength (Figure 3). Figure 3A shows that the first axis explained 19% of variability, splitting the total microbiota (16S rRNA gene) of the patients that were medicated with bactericidal AB (patients A, C and D) from the one treated with a bacteriostatic AB (patient B). The second axis explained 12% of variability, placing the two groups of samples treated with cell replication inhibitor (patient A) and protein synthesis inhibitor (patient B) antibiotics on one side of the graph; these ABs inhibit both essential and related cellular processes, such as DNA replication and protein synthesis. By contrast, the samples from patient C, treated with a cell envelope synthesis inhibitor AB affecting synthesis of the bacterial cell wall, fell on the other side of the graph. Both variables (antimicrobial effect and mode of action) introduced significant variance in the microbiota composition (Adonis test: p =  0.02, p =  0.04, respectively).

Regarding active microbiota (Figure 3B), the first and second axes explained 12% and 6% of the total variability, respectively. With respect to the first axis, the samples from patient B (protein synthesis inhibitor antibiotic) situated on the right side of the graph. The latter AB, as occurred for the total microbiota, introduced higher variance. The second axis separated the samples taken from patients C and D, treated with cell envelope synthesis inhibitor AB from the rest. Despite both factors, the antimicrobial effect and mode of action were not significant (Adonis test: p =  0.18, p =  0.069), the second explained more variability (Adonis test, p =  0.069).

The CCA applied at gene level (Figure 3C) showed a distribution similar to that found for the total microbiota (Figure 3A), with the first and the second axes explaining 25% and 6% of the total variance, respectively. The samples are separated chiefly by antimicrobial effect and then by mode of action. However, in this case the strength of the different vectors is weaker, probably due to the great functional homogeneity of the gut microbial community (Adonis test: antimicrobial effect p =  0.27, mode of action p =  0.41).

Finally, we performed a CCA using the taxonomy of the identified coding regions (Figure 3D). The two axes explained 19% and 7% of the total variation of the data. As can be seen, the different classes of antibiotics affected the taxonomy of the identified coding regions in a similar way to the results reported for total microbiota (Figure 3A) and gene analysis (Figure 3C) (Adonis test: antimicrobial effect p =  0.044, mode of action p =  0.049).

### Functional analysis of the metagenomes

The functional annotation of the ORFs was derived using the TIGRFAM database, providing a hierarchical order: main roles, the highest functional level (described in Figure S2), sub-roles, more specific metabolic functions for each one of the main roles and genes [36]. Regarding the main roles, the profiles were fairly homogeneous for all patients and time points. The most abundant categories before, during and after treatment were “Protein synthesis”, “Energy metabolism”, “Cellular processes” and “Transport and Binding Proteins” with average values of relative abundance of 13.5%, 13.2%, 9.6% and 9.5%, respectively, which highlights the importance of these functions performed by the gut microbiota (Figure S2). However, when considering the sub-roles, we detected significant changes in the corresponding profiles for each patient before, during and after treatment. For all patients we detected a total of 53 sub-roles that differed significantly in gene content (Table 2). Only two sub-role functional categories changed significantly in all patients: “Menaquinone and ubiquinone” (within the main function “Biosynthesis of cofactors, prosthetic groups, and carriers”) and “Carbohydrates, organic alcohols, and acids” (within the main function of “Transport and binding proteins”). Genes participating in the biosynthesis of menaquinone and ubiquinone were under-represented during the treatment for all patients, except in the case of patient B. Regarding the sub-role of “Carbohydrates, organic alcohols, and acids” the gene functions were overrepresented during the treatment in patients A, B and C and under-represented in patient D. Most of the genes belonging to this functional group were related to the phosphotransferase system (PTS), which is essential for translocating carbohydrates in bacteria [39]. Within this family, we have found genes involved in the transport of various sugars such as mannose, fructose, sorbose, glucitol or glucose. It is noteworthy that the related sub-role “PTS” (within the main role “Signal transduction”), associated to genes participating in regulation, was also over-represented during the treatment in patients A and C and under-represented in patient D.

**Table 2:** Functional profiles.

		Patient			
Main Role	Sub-Role	A	B	C	D
Amino acid biosynthesis	Glutamate family	↑ 2.06E-004	NS	NS	NS
	Histidine family	↑ 0.02	NS	NS	NS
	Serine family	↑ 4.36E-004	NS	NS	↑ 0.01
Biosynthesis of cofactors*	Biotin	NS	↑ 0.02	NS	↓ 2.33E-004
	Glutathione and analogs	NS	↑ 2.95E-003	NS	↓ 0.04
	Menaquinone and ubiquinone	↓ 0.02	↑ 2.19E-005	↓ 0.04	↓ 4.68E-003
	Molybdopterin	NS	NS	NS	↓ 4.35E-003
	Pantothenate and coenzyme A	↑ 0.01	NS	NS	NS
	Other	↑ 1.33E-004	NS	NS	NS
Cell envelope	Biosynthesis and degradation of surface**	↓ 0.05	↑ 3.01E-005	NS	↓ 0.02
	Other	↑ 0.01	↓ 0.01	NS	↑ 0.02
	Surface structures	NS	NS	NS	↑0.04
Cellular processes	Biosynthesis of natural products	NS	NS	NS	↓0.05
	Cell division	NS	↓ 0.01	NS	↑ 3.07E-004
	Chemotaxis and motility	NS	NS	NS	↑0.01
	Detoxification	NS	NS	NS	↓ 0.04
	DNA transformation	↓ 2.32E-003	NS	↓ 0.01	NS
	Pathogenesis	↓ 9.43E-004	↑ 1.47E-005	NS	↓ 2.22E-009
	Sporulation and germination	↑ 0.03	↓ 7.07E-017	NS	↑ 2.65E-017
	Toxin production and resistance	NS	↓ 2.85E-004	NS	NS
Central intermediary metabolism	Amino sugars	NS	NS	NS	↑ 0.03
	Nitrogen metabolism	NS	↑ 0.02	NS	NS
DNA metabolism	Chromosome-associated proteins	NS	NS	↓ 0.01	NS
	Restriction/modification	↑ 0.02	NS	NS	NS
Energy metabolism	Aerobic	↓ 0.01	NS	NS	↓ 0.01
	Amino acids and amines	NS	↑ 9.26E-007	NS	↓ 2.22E-009
	Anaerobic	NS	NS	NS	↓ 4.80E-005
	Biosynthesis and degradation of polysaccharides	↓1.58E-003	NS	NS	NS
	Chemoautotrophy	NS	↓0.01	NS	NS
	Electron transport	NS	↑ 0.01	NS	NS
	Entner-Doudoroff	NS	↑ 2.85E-004	NS	↓ 0.01
	Fermentation	NS	NS	NS	↑0.01
	Pentose phosphate pathway	NS	↑ 0.01	NS	↓ 0.03
	Sugars	NS	↑ 2.47E-005	NS	↓ 0.04
	TCA cycle	↑ 0.05	↑ 0.02	NS	↓ 4.59E-003
Fatty acid and phospholipid metabolism	Degradation	↓ 0.05	↑ 0.04	NS	↓ 0.03
Protein fate	Protein and peptide secretion and trafficking	NS	NS	NS	↓ 0.02
	Protein folding and stabilization	NS	↓ 0.01	NS	NS
Protein synthesis	Other	↓ 0.03	↓ 4.57E-005	NS	NS
	Ribosomal proteins: synthesis and modification	NS	↓ 2.85E-004	NS	↑ 4.80E-005
	tRNA and rRNA base modification	NS	NS	↑ 2.04E-003	NS
Regulatory functions	Other	NS	NS	↑ 0.01	NS
Signal transduction	PTS	↑ 1.40E-005	NS	↑ 3.59E-009	↓ 7.09E-009
	Two-component systems	NS	NS	NS	↓1.52E-003
Transcription	DNA-dependent RNA polymerase	NS	↓ 4.49E-003	NS	↑ 0.03
Transport and binding proteins	Amino acids, peptides and amines	↑ 4.36E-004	↑ 2.85E-004	NS	↓ 4.32E-005
	Anions	↓ 4.36E-004	NS	NS	NS
	Carbohydrates, organic alcohols, and acids	↑ 4.36E-004	↑ 3.62E-006	↑ 1.46E-004	↓ 3.76E-033
	Cations and iron carrying compounds	↑ 3.03E-003	NS	NS	↓ 0.04
	Nucleosides, purines and pyrimidines	NS	↑ 2.61E-003	NS	↓ 1.52E-003
	Other	NS	↑ 1.33E-003	NS	↓ 5.56E-004
	Porins	NS	↑ 0.01	NS	NS
Unknown function	Enzymes of unknown specificity	↓ 0.04	NS	NS	↓4.97E-005

Main roles and sub-roles that change significantly during treatment and their associated p-values (p-value < 0.05). The upward arrow indicates those categories that were more abundant during treatment and the downward arrow those that were less abundant. NS, not significant.

* Biosynthesis of cofactors, prosthetic groups, and carriers.

** Biosynthesis and degradation of surface polysaccharides and lipopolysaccharides.

The changes in the sub-roles “Biosynthesis and degradation of surface polysaccharides and lipopolysaccharides” and “Other” (from the “Cell envelope main role”) were significant in patients A, B and D. In general, we detected a lower presence during treatment of genes involved in the synthesis of lipopolysaccharides (LPS). The sub-role “Pathogenesis” (within the main category “Cellular processes”) and “Degradation” (within the “Fatty acid and phospholipid metabolism” main category) decreased significantly during treatment for patients A and D and increased in patient B. The fatty acid and phospholipid metabolism genes were involved in fatty acid beta-oxidation. On the contrary, the sub-role “Sporulation and germination” (within “Cellular processes”) was more abundant during treatment in patients A and D, with most of the genes being involved in different stages of endospore formation. Finally, we found that the sub-role “TCA cycle” (within the main category “Energy metabolism”) and “Amino acids, peptides and amines” (within “Transport and binding proteins) underwent an increase in the number of genes encoding different enzymes of the citric acid cycle during antibiotic treatment for patient A and B and a decrease for D. Regarding the transport of amino acids, peptides and amines, we found the presence of genes encoding ABC transporters for amino acids and urea.

### Analysis of the resistome

**Figure 4:**
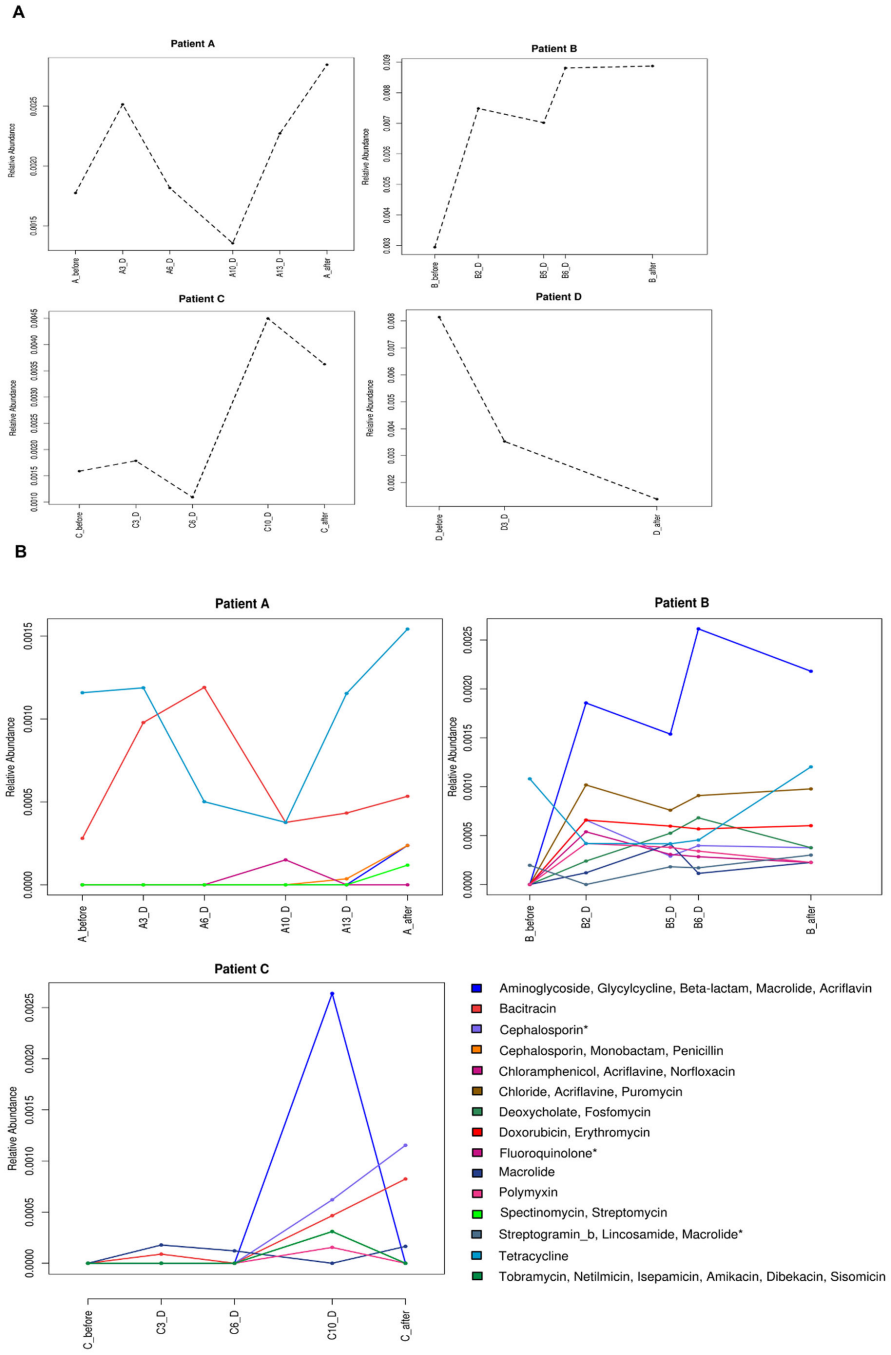
Resistance gene profiles. (A) The dashed lines represent the relative abundance of the total number of resistance genes for patients A, B, C, and D. (B) Relative abundance of the resistance genes throughout AB treatment for patients A, B, and C. The symbol "*" highlights the resistance gene profiles which coincide with the antibiotic administered to patients C, A and B, respectively.

We performed a search of the resistome by identifying AB resistance genes in the 19 metagenomes analyzed by comparing the predicted ORFs against the Antibiotic Resistance Genes Database [37]. We identified the resistance genes that represented 0.2%, 0.8%, 0.22%, and 0.5% of the total determinants found for patients A, B, C and D respectively. We found that while patients A, B, and C showed an increase in resistance genes after treatment, patient D presented the lowest relative abundance of these determinants, decreasing from 0.81% to 0.14% (Figure 4A). Figure 4B shows the profiles of resistance genes that varied during the treatment for patient A, B and C, administered with antibiotics belonging to different classes: fluoroquinolone, lincosamide and beta-lactams, respectively (Table 1). Overall, we observed that the resistance induced by each antimicrobial was associated with other resistance determinants. Also, we found that its profiles matched fairly well with the oscillatory dynamics of the surviving bacterial community. Patient A showed an increase in the relative abundance of the total resistance genes at the end of the treatment, raising values from 0.18% before treatment to 0.28% after the AB course. Fluoroquinolone resistance, multidrug resistance efflux pump, appeared on day 10, when the microbiota composition was dominated by the Firmicutes phylum, with high abundance of members of the genus *Enterococcus*, described as resistant to this type of antimicrobial [40]. This patient presented high relative abundance of *tetq* gene, which confers tetracycline resistance before and after the treatment, being *Bacteroides* genus one of the most abundant taxa. Bacitracin profile showed a maximum on day 6, with *baca* gene being associated to *Streptococcus* and Clostridiales taxa (Figure 1 and 2). Patient B showed a strong increase in the relative abundance of resistance genes during treatment, increasing from 0.29% up to 0.89%. In fact all the genes increased in abundance after AB treatment except those involved in bacitracin resistance. The most remarkable increase was found in a group of genes coding for multidrug resistance efflux pump, which confer resistance against clindamycin and related antimicrobials (aminoglycoside, glycylcycline, beta-lactam, macrolide, and acriflavine). Patient C also showed an increase in the total relative abundance of resistance genes at the last time point and after treatment, from 0.16% to 0.36%. The genes that code for multidrug resistance efflux pumps are the most abundant on the 10^th^ day of treatment. However, beta-lactamase genes increased throughout the AB course and reached the maximum at the end of treatment. Patient C also presented a high abundance of tetracycline resistance genes before treatment (*teto*, *tetq* and *tetw*) associated to different taxa (*Blautia, Bacteroides, Clostridium, and Ruminococcus*), which have been described as resistant to this antibiotic [41], [42], [43] but underwent a dramatic decrease on day 10 associated with a major presence of the Proteobacteria phylum.

## Discussion

### Dynamics of the gut microbiota structure over the AB course

The human gut microbiota consists of a highly complex community whose members establish close relationships with the host. ABs have strong direct and indirect effects on the human gut microbiota and consequently on the functions they perform, affecting the ecosystem maintenance and therefore host physiology [7], [13]. The microorganisms that carry certain genetic determinants have an advantage under AB pressure, allowing them to survive and grow. It is well known that the human gut microbiota presents a high inter-individual variability and that its composition depends on factors such as genetics, age, diet, health status and AB-therapy, among others.

In our study, each patient presented their own initial microbiota and thus there was an individual response to AB treatment with fluctuations in the bacterial diversity and composition for both total and active gut microbiota. These results highlight the importance of the initial microbial structure in shaping the changes in microbiota during the AB course. The individual character of the response and incomplete recovery of initial microbiota after AB treatment has previously been described by Dethlefsen and coworkers [18] in a follow-up study of three patients that received two courses of ciprofloxacin. However, we also observed that the selection of resistant microorganisms led to a similar microbiota composition after analogous antibiotic treatment. Thus, AB seems to have a major impact on the structure of the final bacterial community.

The gut microbiota has been described as an ecosystem that is relatively resistant to perturbations [16]. However we observed that a particular assembly of microorganisms can confer greater resistance to a disturbance than others in terms of presence and abundance of taxa, which could be related with the specific effect of the AB. In patient A, there was a decrease in *Faecalibacterium* and *Bacteroides* genera during the AB course, with AB-resistant strains appearing at the end of treatment. However, on the first days of treatment, other butyrate-producing taxa (*Roseburia* and *Lachnospiraceae incertae sedis)* and H2-consuming bacteria (*Blautia*, *Collinsella* and *Bifidobacterium*) were present as active microbiota, obtaining energy sources for the colonocytes of the host. A similar behavioral pattern of these members of the intestinal microbiota has also been reported by the above mentioned group of Dethlefsen et al. [15], [18] when they used ciprofloxacin, as in our patient A, an AB belonging to the cell replication inhibitor group. In the case of patient B, since clindamycin affected anaerobic bacteria, there were marked decreases in *Bacteroides* and *Blautia* genera in the active microbiota just after AB administration (Figure 2B). However, three days later, high abundances of *Bacteroides* were detected in the active bacterial community, suggesting these bacteria acquired resistance. The presence of clindamycin-resistant *Bacteroides* in gut microbiota has also been described in other studies [9], [44]. Moreover, we observed that the decrease in anaerobic bacteria is compensated for by an important increase in members of the family Enterobacteriaceae. Patients C and D were treated with ABs which have a similar mode of action as both are of the β-lactam class. As stated, the initial microbiota composition was very different between both subjects, showing a differential response to ABs. However, the active microbiota changed throughout AB treatment with both patients acquiring a similar composition by the end of it (Figure 2B). Patient C received a combination of two ABs, Cefazolin and Ampicillin/Sulbactam, which cover a broad spectrum of microorganisms and showed a significant increase in *Parabacteroides* and *Bacteroides* genera. Interestingly, resistance genes against ampicillin and cephalosporin in these two taxa have been described previously [43]. On day 10 of treatment, an increase in the Enterobacteriaceae family occurred and some of its genera, such as *Escherichia* or *Klebsiella* are considered as opportunistic pathogens [45], suggesting that AB use creates opportunistic infections by these harmful microorganisms. Patient D was treated with amoxicillin, described as active against some Proteobacteria such as *Escherichia* or *Klebsiella*. During treatment, these genera were almost eliminated, whereas *Bacteroides*, *Blautia* and *Faecalibaterium* taxa proved less susceptible to treatment, as occurred in patient C. It is worth pointing out that this bacterial profile, with *Bacteroides*, *Blautia* and *Faecalibaterium* after the AB stress, has been found in the case of bactericidal agents but not when a bacteriostatic antimicrobial was used. Furthermore, a previous study [12] found a similar pattern of active bacteria after beta-lactam treatment.

### Antimicrobial effect and mode of action of ABs on the gut microbiota

It has been stated that external variables such as ABs shift the microbial composition [46]. In our study, the class of AB significantly shaped the microbiota on the basis of the antimicrobial effect (bactericidal or bacteriostatic) and the mode of action. In addition, we have found that specific mechanisms of action affect some organisms more than others, leading the bacterial community towards an alternative temporary equilibrium state. Clindamycin (protein synthesis inhibitor) introduced higher variance in microbiota composition than the other agents, giving way to a different bacterial community structure. This was probably due to the bacteriostatic nature of clindamycin when compared to the bactericidal effect of the other AB treatments. Interestingly, in the case of the active microbiota representing the surviving community, the bacterial composition was affected by the mode of action rather than the antimicrobial effect clearly distinguishing the three modes of action (Figure 3B). At a functional level, the microbial community profile was driven by the antimicrobial effect rather than by the mode of action. However, the strength of the AB class, considered as an external factor exerted at gene level was less intense, resulting in major uniformity.

### AB impact on bacterial metabolic functions

High homogeneity was observed in the main roles for all the patients. This uniformity at a functional level has been also shown in both DNA and RNA-based surveys [22], [47], [48], [49], [50] since the microbiota is characterized by high functional redundancy. When we considered sub-roles, 51% with significant variation corresponded to inter-individual variability, representing the specific-subject response to AB course. The over-representation of genes involved in sugar transport in most of the patients suggested that this functional category could play an important role under stress conditions, as is the case of AB treatment. The phosphotransferase system (PTS), in addition to its main role in sugar transport, which is an essential function in itself, is involved in different regulatory processes such as stress response in bacteria and, hence, it could confer some extra advantages in presence of ABs [51]. Then, an efficient system of importing sugars could facilitate the energetic metabolism and, therefore, it could counteract the negative effect of ABs on the bacterial growth. Pérez-Cobas and coworkers [12] showed an increase in proteins belonging to the glycolysis pathway and pyruvate metabolism, as well as higher expression of genes related to energy metabolism/sugars category during beta-lactam treatment.

As we mentioned previously, the bacteriostatic effect drives the bacterial community to a characteristic composition, which is also reflected at a functional level. In patient B, most of the functional categories over-represented during treatment could be related with the increase in Enterobacteriaceae members. In this regard, we found an increase in the number of genes involved in lipopolysaccharide synthesis, which is the main component of the outer membrane for most Gram-negative bacteria. This barrier plays an important role in nutrient uptake and also confers resistance against ABs [52]. Likewise, the genes of secretion systems typical of Gram negative bacteria pathogenesis showed an increase only in patient B. However, patients A, C, and D, who received bactericidal treatment, presented a high abundance of Gram positive Ruminococcaceae and Lachnospiraceae families, and an over-representation of genes involved in endospore formation, a resistance mechanism typical of Gram positive bacteria. Another category that presented differences between bactericidal and bacteriostatic ABs was catabolism of fatty acids and phospholipids, to produce acetyl-CoA through the beta-oxidation process. As clindamycin inhibits mainly the anaerobic bacteria, the genes belonging to this sub-role were more abundant in patient B whose bacterial composition proved rich in Enterobacteriaceae family members.

### Changes in the resistome

It has previously been pointed out that the AB usage is the most influential agent in the spread and stabilization of resistance genes in the gut environment [13]. One of the multidrug resistant genes that increased in patients A and B was a multidrug resistance efflux pump, which confers resistance against aminoglycoside, glycylcycline, beta-lactam, macrolide and acriflavine antibiotics. Since these ABs have different properties such as spectrum or mode of action, the transmission of these genes to a pathogen could hinder clinical treatment in the event of infection. These resistance genes have been described in some Proteobacteria genera such as *Escherichia* or *Klebsiella*, thereby supporting the increase in the abundance of these genera during treatment, principally in patient B. Patients A, B and C reached higher values of gene resistance abundances after AB treatment, with patient B, who was treated with clindamycin, attaining maximum values. It has been reported that besides the strong effect on the microbial composition, clindamycin also promotes increased AB resistance, which can persist in the microbial population for a long time [53]. In contrast, patient D showed a decrease in the relative abundance of resistance genes in the bacterial community. In fact, we found different dynamics in patient D as compared to the other three patients. This sample presented an initial composition with prevalence of the Enterobacteriaceae family, which has been described as a considerable source of resistance genes [54] and hence, the data indicated that these taxa were strongly affected by the ABs. Thus, the final resistome in the human gut after AB therapy would be determined by the resistance genes carried by the surviving bacteria and by the class of AB administered.

## Conclusions

In this study, using high-throughput methodology, we have provided new insights into the complex antibiotic resistance scenario, related to the different modes of action of antibiotics and the consequences for the gut microbiota composition and function during antibiotic therapy. We have shown that specific properties of ABs such as antimicrobial effects or mode of action, are powerful forces for the selection of intestinal microbiota, and are partially responsible for the shifts in bacterial composition during AB therapy. The resulting structure of the microbial community showed its specific metabolic capabilities giving a different functional profile. Additionally, we have shown that the AB also modified the resistome composition, increasing the abundance of resistance genes in the gut environment, which is also important in shaping the post-treatment composition of the microbiota. However, further research into a larger group of subjects would be necessary to establish a quantitative evaluation of changes in gut microbiota.

## Supporting Information

Figure S1 **Evolution of diversity parameters along the treatment for patient A, B, C, and D.** (A) Shannon Index. (B) Richness estimators: N, Chao1 and ACE. N is the number of observed taxa.
(TIFF)

Figure S2 **Abundance of the main functional roles for all the samples.**
(TIFF)
