# Supplementary figures and images for: Differential Effects of Antibiotic Therapy on the Structure and Function of Human Gut Microbiota
Source: PLoS One. 2013 Nov 25;8(11):e80201. doi: 10.1371/journal.pone.0080201 (PMC3839934; doi:10.1371/journal.pone.0080201)

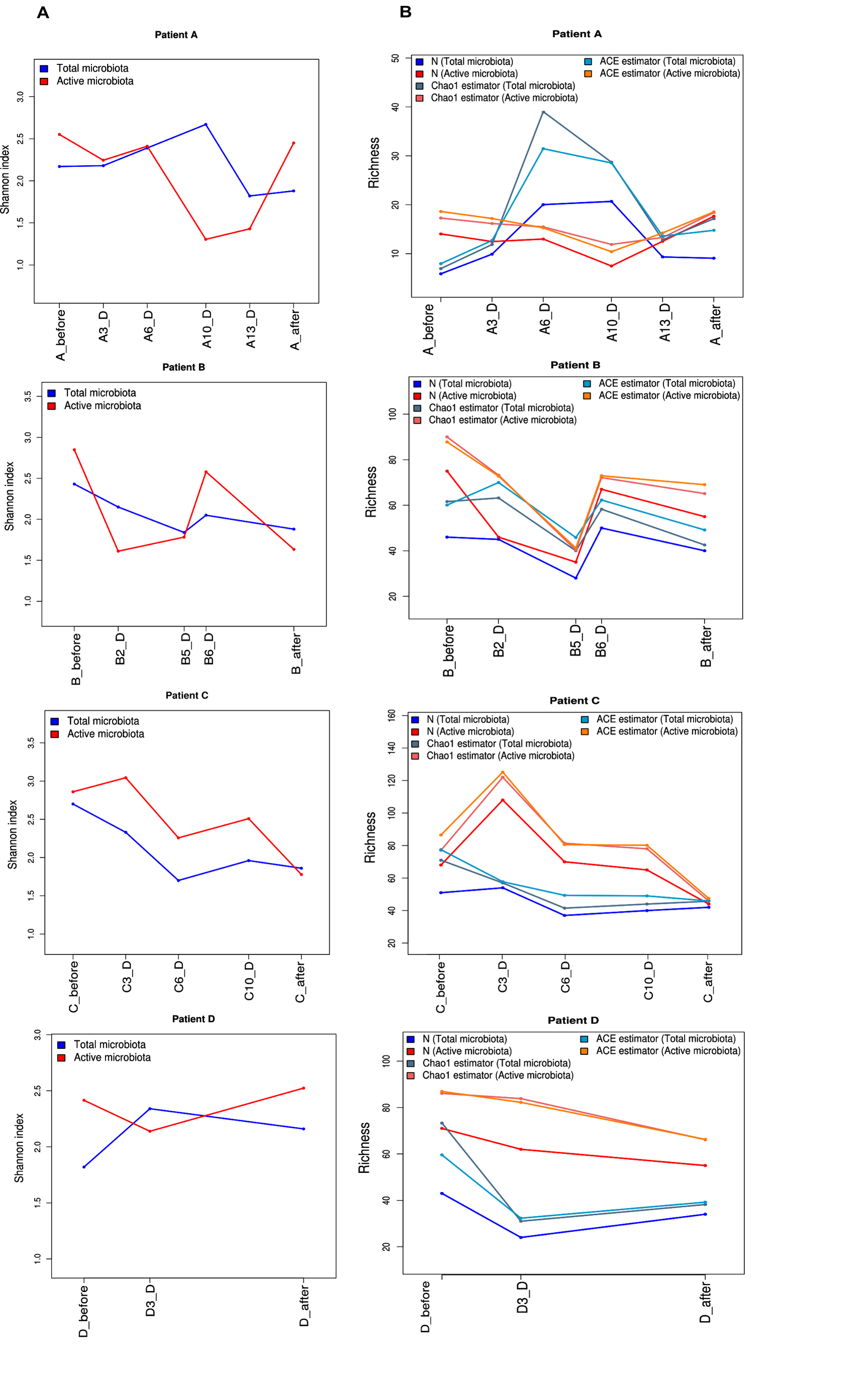

Supplement: Figure S1 — Evolution of diversity parameters along the treatment for patient A, B, C, and D. (A) Shannon Index. (B) Richness estimators: N, Chao1 and ACE. N is the number of observed taxa. (TIFF) [file pone.0080201.s001.tiff]

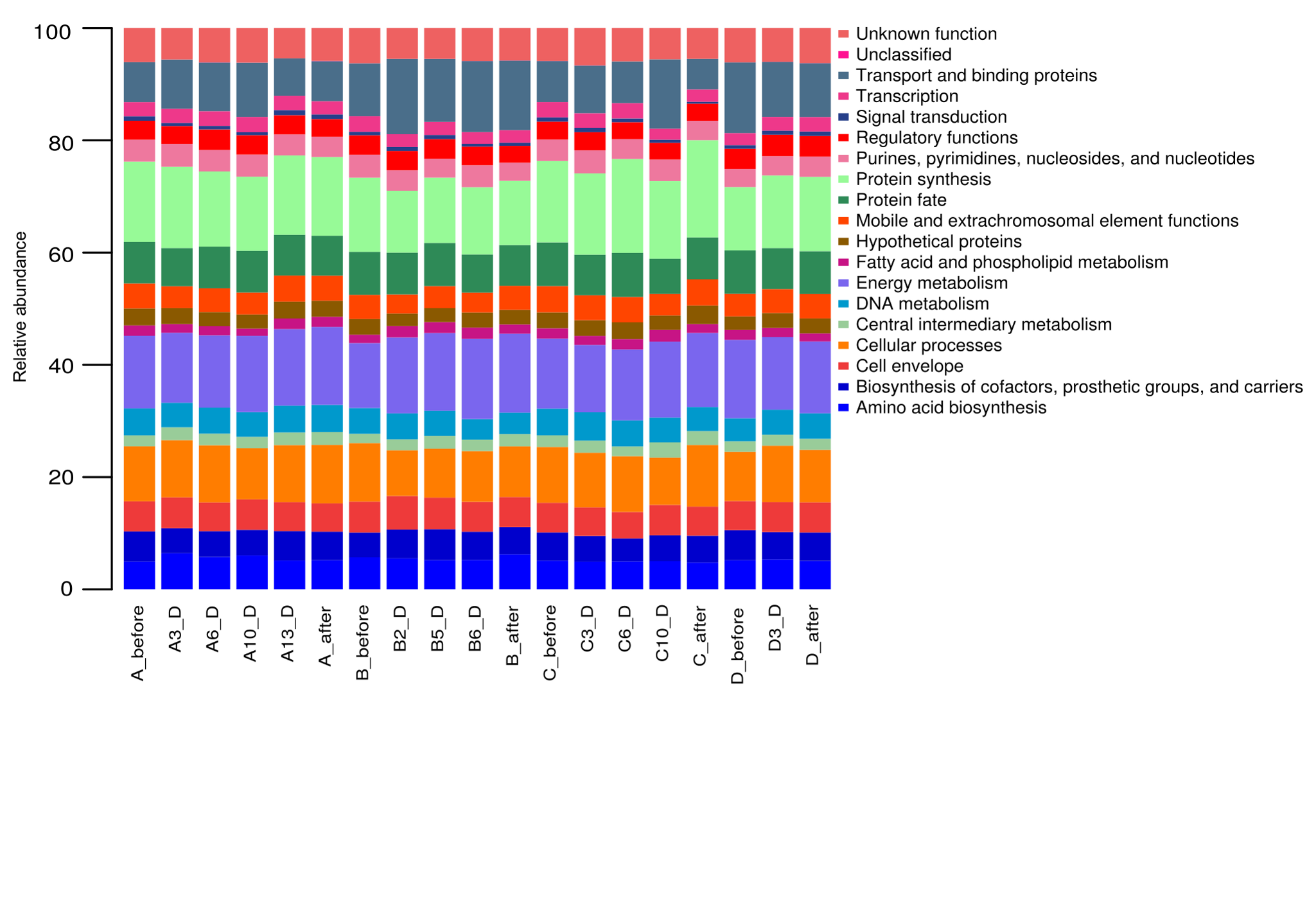

Supplement: Figure S2 — Abundance of the main functional roles for all the samples. (TIFF) [file pone.0080201.s002.tiff]
